# An Overview of Short-Bowel Syndrome in Pediatric Patients: Focus on Clinical Management and Prevention of Complications

**DOI:** 10.3390/nu15102341

**Published:** 2023-05-17

**Authors:** Chiara Caporilli, Giuliana Giannì, Federica Grassi, Susanna Esposito

**Affiliations:** Pediatric Clinic, Department of Medicine and Surgery, University of Parma, 43126 Parma, Italy

**Keywords:** intestinal adaptation, intestinal failure, parenteral nutrition, short-bowel syndrome, small intestinal bacterial overgrowth

## Abstract

Short-bowel syndrome (SBS) in pediatric age is defined as a malabsorptive state, resulting from congenital malformations, significant small intestine surgical resection or disease-associated loss of absorption. SBS is the leading cause of intestinal failure in children and the underlying cause in 50% of patients on home parental nutrition. It is a life-altering and life-threatening disease due to the inability of the residual intestinal function to maintain nutritional homeostasis of protein, fluid, electrolyte or micronutrient without parenteral or enteral supplementation. The use of parenteral nutrition (PN) has improved medical care in SBS, decreasing mortality and improving the overall prognosis. However, the long-term use of PN is associated with the incidence of many complications, including liver disease and catheter-associated malfunction and bloodstream infections (CRBSIs). This manuscript is a narrative review of the current available evidence on the management of SBS in the pediatric population, focusing on prognostic factors and outcome. The literature review showed that in recent years, the standardization of management has demonstrated to improve the quality of life in these complex patients. Moreover, the development of knowledge in clinical practice has led to a reduction in mortality and morbidity. Diagnostic and therapeutic decisions should be made by a multidisciplinary team that includes neonatologists, pediatric surgeons, gastroenterologists, pediatricians, nutritionists and nurses. A significant improvement in prognosis can occur through the careful monitoring of nutritional status, avoiding dependence on PN and favoring an early introduction of enteral nutrition, and through the prevention, diagnosis and aggressive treatment of CRSBIs and SIBO. Multicenter initiatives, such as research consortium or data registries, are mandatory in order to personalize the management of these patients, improve their quality of life and reduce the cost of care.

## 1. Introduction

Short-bowel syndrome (SBS) is a complex disease that occurs due to the physical loss or the loss of function of a portion of the small and/or large intestine [1]. Consequently, individuals with SBS often have a reduced ability to absorb nutrients such as fats, carbohydrates (sugars), vitamins, minerals, trace elements and fluids (malabsorption). In pediatric age, SBS results from congenital malformations, significant small intestine surgical resection or disease-associated loss of absorption. It is the leading cause of intestinal failure (IF) in children and the underlying cause in 50% of patients on home parental nutrition (HPN) [2]. The severity of malabsorption is determined by the length and quality of the remaining bowel, necessitating a varying degree of support with enteral and parenteral nutrition (PN) [3,4].

Intestinal adaptation is the process by which the gastrointestinal remnant tract responds to fit the physiologic needs of the patient [5,6]. It concerns morphologic and functional changes aimed at gradually increasing absorptive capacity. Recent advances in our knowledge of the underlying pathophysiology in SBS have improved these patients’ ever-evolving care. The therapeutic approach includes a complete evaluation concerning nutritional, medical and surgical aspects by a multidisciplinary team [5,6].

The use of PN has improved medical care in SBS, decreasing mortality and improving the overall prognosis [3]. However, the long-term use of PN is associated with the incidence of many complications, including liver disease and catheter-associated malfunction and bloodstream infections [3]. The multidisciplinary intestinal rehabilitation programs (IRPs) shape the management of these patients. The key is complication mitigation, which involves focusing on avoiding catheter complications, infections, nutrient deficiencies, metabolic disorders and cholestatic liver disease. In the meantime, the IRP management is aimed at promoting intestinal absorption and gaining enteral autonomy [7]. This approach has changed the prognosis, thus limiting the nutritional failure, which is considered a major indication for intestinal transplantation.

This manuscript is a narrative review of the current available evidence on the management of SBS in a pediatric population, focusing on prognostic factors and outcome. From results obtained in recent years, in fact, the standardization of management has demonstrated to improve the quality of life in these complex patients. We focused on the latest literature available, so we decided to look for papers not older than 12 years, i.e., published in 2010 or after. Some articles published before 2010 were included in the bibliography because of their importance for the topic. We searched up to December 2022 in several databases, namely Pubmed, Cochrane Library, Embase, Web of science, Google Scholar and Scopus, up to December 2022. Keywords included words and expressions related to the topic, such as “short bowel syndrome (SBS)”, “intestinal failure (IF)”, “pediatric”, “parenteral nutrition (PN)”, “intestinal adaptation”, “liver disease”, “sepsis”, “small intestinal bacterial overgrowth (SIBO)”, “catheter-related complications”, “D-lactic acidosis”, “IFALD”, “child”, “children”, “pediatric”, “intestinal rehabilitation program”, “therapy” and “treatment”. Additional papers have then been identified through the references of the chosen literature. More than 1500 manuscripts were identified, but only those published in English with original data or systematic reviews were considered.

## 2. Epidemiology

Pediatric epidemiological data about the exact prevalence or incidence of intestinal failure are fragmentary. The accurate estimate of SBS incidence is difficult to determine because of the rarity of the condition, the variability of the definition of SBS in different institutions and the difficulty of tertiary care centers in the selection of the catchment population. Cole et al. published data referring to the incidence of SBS in a large cohort of very low birth weight infants from a selection of 16 tertiary neonatal centers in the United States [8]. Estimates showed that the incidence of surgical SBS was 0.7% (7 per 1000) among 12,316 very low birth weight infants, and was 1.1% (11 per 1000) among 5.657 extremely low birth weight infants. Moreover, 96% of these had been caused by necrotizing enterocolitis. These data do not consider term neonates in which congenital malformations are significantly relevant for SBS etiology. Regrettably, the SBS malabsorptive state was not defined beyond a need for PN support after a massive surgical resection of the gastrointestinal tract [8].

In 2008, the Italian Society of Paediatric Gastroenterology Hepatology and Nutrition and the Italian Society of Neonatology showed data deducted from seven tertiary neonatal intensive care units over 2003 and 2004 [9]. SBS was defined as a residual gut length less than 25% of that predicted for gestational age or as the need for PN for more than 42 days after intestinal loss of function or surgical resection. The incidence of IF was described as 0.1% in all live births (26/30,353) and 0.5% (26/5088) among neonates belonging to the neonatal intensive care unit [9].

Referring to actual literature evidence, a single large population-based study marks estimates examining SBS incidence and mortality. This Canadian study considered a current definition of SBS based on the need for PN for greater than 60 days after intestinal surgical resection or a loss of gut length of less than 25% of that expected [10]. The authors referred to administrative health care data and federal census data from a selected catchment area. The overall rate of SBS was 24.5 per 100,000 live births (95%CI = 12.1, 36.9), with a greater incidence in preterm infants. The main causes in the neonatal group were necrotizing enterocolitis (35%) and complicated meconium ileus (20%) [10].

The functional definition of SBS considers the critical loss of the length of gut mass or its function below a minimum required for the adequate absorption of enteral nutrients and fluids to provide essential nourishment for the growth and the maintenance of life [7]. SBS severe forms account for 44% of the cases [11,12,13]. However, home PN is not only offered to children with SBS, but also to those who have IF due to a different etiology [14].

## 3. Etiology

In pediatric age, the conditions mainly involved in SBS etiology are congenital and perinatal diseases, such as necrotizing enterocolitis (NEC), malrotation leading to midgut volvulus, abdominal wall defects (gastroschisis) and intestinal atresia, while long segment involved Hirschprung’s disease and extensive aganglionosis are more rare causes [15]. However, NEC itself causes almost one third of all the reported cases. Referring to the neonatal period, acquired SBS secondary to NEC is the most represented age. The number of these patients increases among preterm infants, with less surface for intestinal absorptive function; in addition, in infants with ischemic intestinal events, an increased rate of NEC is estimated [15].

Overall, 20% of SBS develops in the non-neonatal population, relating to different causes, such as volvulus and trauma. In young adults, rarely in children, Crohn’s disease, leading to multiple massive bowel resections, is the most frequent cause of SBS [3]. Less prevalent etiologies of SBS in pediatric populations are mesenteric infarction related to thrombotic disease and cancer, which is seen more frequently in adults than in children [14,16].

## 4. Intestinal Rehabilitation Programs

Pediatric patients who suffer from SBS are heterogeneous and complex, requiring coordinated, timely and intensive medical care. The management of pediatric SBS is based on collaborative supporting activities. One of the keys to the management of SBS is multidisciplinary treatment, including pediatricians, gastroenterologists, surgeons, nutritionists, nurses, social workers and psychological supporters. These programs set the focus on obtaining an adequate feeding profile and minimizing the onset of complications through careful clinical monitoring and reasoned use of medical and surgical therapies [17,18,19]. The attention is on the formulation of a strategic nutritional supply to integrate the absorption of enteral nutrients; it is crucial to undertake an aggressive approach to hepato-protective strategies to minimize intestinal failure-associated liver disease, prevent catheter-related bloodstream infections, small bowel bacterial overgrowth, nephrocalcinosis and metabolic bone disease [20].

The increasing experience of allied health professional teams allowed for a dramatic improvement in the outcomes of patients affected with SBS. The available literature reports that the introduction of intestinal rehabilitation programs (IRP) is associated with the improved survival and achievement of early weaning from PN [19,21,22,23]. Clinical guidelines from the American Society for Parenteral and Enteral Nutrition suggest that IRPs positively influence prognosis and reduce morbidity and mortality [24,25]. The management of SBS in children in consultation with intestinal rehabilitation centers is also encouraged by the North American Society for Pediatric Gastroenterology, Hepatology and Nutrition [12]. Multidisciplinary teams need effective and timely updates based on a current understanding of the ever-evolving care of these patients in order to refine the clinical management, promote enteral autonomy, recognize complications and support these frail and complex patients and their family.

## 5. Intestinal Adaptation 

After massive bowel loss, the gastrointestinal tract responds with a process called intestinal adaptation, which consists of physiologic changes by the remaining bowel, in intestinal morphology and function, occurring in order to gradually improve the absorption of adequate nutrients and fluid and recover part of the residual functionality. In pediatric patients, this process begins shortly after gut loss and continues for several years, both on the macroscopic and microscopic levels [1]. The first period is characterized by diarrhea and a massive loss of fluids and electrolytes; the use of total PN is often suggested. In the following months, the remaining bowel gradual modifications are seen [20].

Already in the initial stages, epithelial hyperplasia occurs, which includes the lengthening of residual villi and deepening of the crypts; furthermore, microvilli proliferation is observed [26]. Anatomical changes are demonstrated by the dilatation and elongation of the remnant bowel; bowel wall thickness improves, due to the hypertrophy of smooth muscle layers. An increase in intestinal weight and the total enterocyte number occurs [26]. 

As demonstrated, changes in gene expression permits significant increases in villus height, crypt depth and the crypt proliferative and apoptotic index; recent findings suggest that enterocyte proliferation during adaptation is regulated by EGFR signaling in cells other than enterocytes, perhaps within the mesenchymal cell compartment of the bowel wall [27,28]. Besides enhanced enterocyte proliferation, it has been revealed that adaptation is associated with angiogenesis. In fact, prominent angiogenesis leads to improved blood circulation in the gastrointestinal tract [29,30,31].

A more adaptive capacity of the ileum compared to the jejunum is well-documented [32,33,34]. Structural and functional changes are more prominent in the ileum than in the jejunum. Adaptation in the jejunum is functional rather than structural and is associated with transporter and enzyme activity. On the microscopic side, trans-membrane transporters are upregulated, causing changes in the fluidity and permeability of the brush border membrane [32,33,34]. After the initial months, moderate enteral nutrition (EN) can be initiated. In the subsequent phases, EN is (i.e., feeding with a feeding tube) one of the more effective stimulants of intestinal adaptation [35]. Gradually, feeding via EN is promoted and PN is weaned [36]. 

Pharmacological and nutritional agents are classified considering their effective role on absorptive capacity, intestinal adaptation and bowel motility, the three major strategies employed in the management of SBS [37]. Nutritional strategies include soluble fiber, enteral fat, complex proteins, glutamine and probiotics [38,39]. Sodium supplementation influences the diarrhea symptoms, the pancreatic-biliary function and actually favors the intestinal adaptation process. Specifically, fatty acids encourage intestinal adaption by stimulating the production of gastrointestinal hormones [38,39]. Recent studies considering the role of the glucagon-like peptide 2 (GLP-2), an intestinal growth factor produced by the L cells of the ileum and colon, have led to the production of teduglutide, i.e., a long-acting GLP-2 analogue used in the pharmacological approach of adults with PN-dependent SBS and in the promotion of intestinal absorption for reducing PN needs in the pediatric population [38,39]. Other hormones have been studied, such as the growth hormone, insulin-like growth factor 1, epidermal growth factor, glucocorticosteroids, thyroid hormone and leptin [27].

Research investigations on the optimal clinical practice to promote intestinal adaptation in infant SBS are scarce, and the little evidence available is consistently of lower quality, resulting in a wide variability of clinical presentation. However, the recent increase in studies in this field shows the effort of the scientific community to fill this gap. Significant research has improved our understanding of the pathophysiology and the way we care for these patients, in order to promote enteral autonomy and improve patient outcomes.

## 6. Prognostic Criteria

In the early stages, prognostic criteria are considered to determine the severity of SBS. Markers of intestinal function and outcome include remnant bowel length, quality of residual bowel, site of small bowel resected, loss of the ileum and/or ileocecal valve and/or colon, potential presence of intestinal continuity and enteral autonomy [6,40]. Remnant bowel length, measured from the ligament of Treitz, is historically described as a critical predictive factor in determining prognosis in SBS [14]. The quantification of normal intestinal length in the pediatric population is discussed because it is variable between different ages, and gut growth potential is more pronounced in children than in adults, in term neonate than in premature ones [14].

From the literature data, small intestinal length in a full-term neonate range has been estimated between 176 and 305 cm, considering discrepancies due to the alterations of smooth muscles, belonging to post-mortem measurements [41]. In general, more recent studies showed that term infants are estimated to have ~150–250 cm of small bowel length; moreover, the length of gut doubles in the third trimester [5]. Studies that account for an absolute measurement (in centimeters) of bowel length, without adjusting for gestational age, should be considered misleading; residual bowel depends on gestational age and should be presented as a percentage of the original total bowel length or of that expected for a particular age [14].

Furthermore, the part of the gut lost affects prognosis [32,33]. The ileum is more adaptable than the jejunum and its preservation is related to a better outcome; the ileum is more able to enhance the villus surface area, in villus height and crypt depth, and to develop processes of increasing the length, diameter and motor function [32,33]. This part of the bowel, compared to the jejunum, is specialized in the absorption of vitamin B12, bile acid and fluid absorption, and is effectively able to increase its absorptive capacity; moreover, the differentiation of the ileal epithelium into the more proximal jejunal epithelium after massive bowel loss has been demonstrated [34]. Moreover, the quality of the remaining bowel, in terms of stenosis or abnormal dilation, has to be considered [1].

Regarding the importance of the preservation of the ileocecal valve (ICV) as a prognostic factor, data are conflicting. The absence of ICV could be a marker of the absence of the resection of ileum, in consideration of their anatomical proximity [42,43]. ICV prevents the passage of small bowel contents into the colon, slowing down intestinal transit time. Its presence also prevents the reflux of colonic contents into the small bowel, limiting the risk of developing small bowel intestinal overgrowth (SIBO); SIBO negatively influences the malabsorptive state, exacerbating diarrhea and malnutrition [44]. The results obtained by Goulet et al. showed a longer duration of PN and a lower weaning rate from PN in children with resected ICV [45].

The patients with a massive small bowel resection, in addition to a resected colon, lose a large part of their gastrointestinal tract, and in particular, they are at major risk of dehydration [46]. In fact, the colon has a slower transit time to retain sodium and water from the lumen; the colon also absorbs nutrients through fermented carbohydrates. Patients affected by SBS with the colic in continuity can take up to 50% of their nutritional requirements via their colon. However, studies have not demonstrated the benefits on enteral autonomy from colon retention [46]. Considering that the preservation of the continuity of the gastrointestinal tract is important to potentiate the mucosal absorptive and digestive capacity, the early closure of stomas promotes the weaning from the PN [1].

The surgical anatomy of the remnant gut and liver function are two further important variables that should determine the selection of therapeutical strategies and prognostic expectations.

## 7. Complications

**Figure 1:**
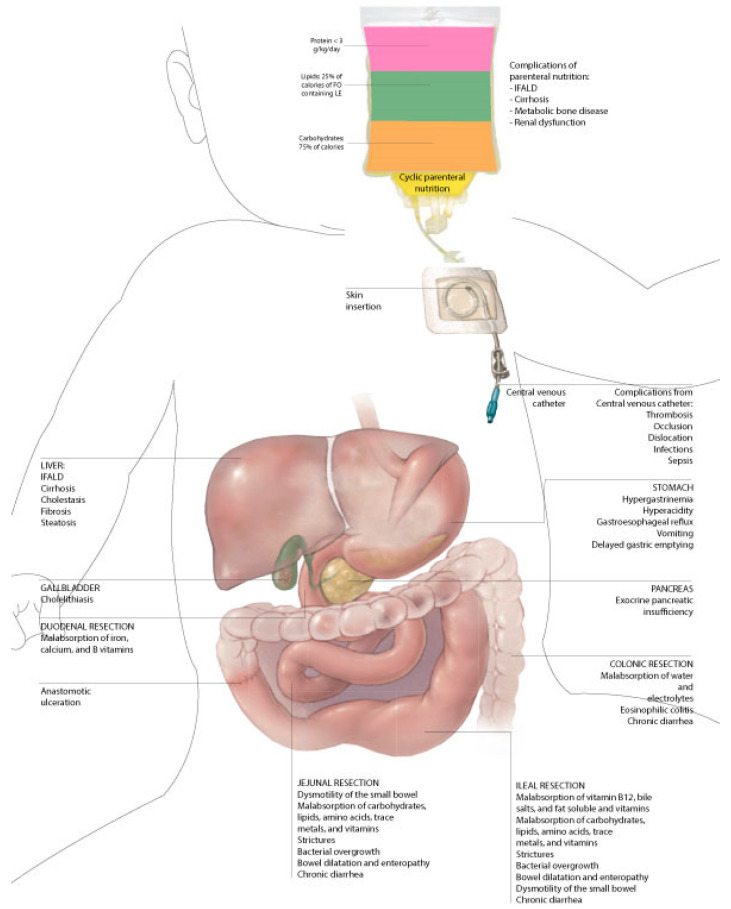
Complications of short-bowel syndrome in children.

Patients affected with SBS are at an increased risk of complications related to the malabsorptive state and the need for long-term PN that affect their quality of life (Figure 1). Patients with irreversible and life-threatening complications become eligible for small bowel transplantation.

### 7.1. Catheter-Related Complications

Catheter-related complications have the greatest impact on long-term outcomes, place the SBS patients at greater risk of death and are a clear key risk factor for morbidity and mortality among patients with IF [12]. Catheter-related complications include occlusions, thrombosis, catheter rupture, dislodgement and sepsis [12].

Central venous thrombosis can lead to a loss of venous access and the subsequent inability to refer the patient to PN, placing the indication for intestinal transplantation. The loss of venous access due to thrombosis is replacing advanced liver disease as a common indication for transplantation [47].

Children with SBS are forced to be on long-term PN, and the incidence of catheter-related bloodstream infections (CRBSIs) ranges from 1.3 to 10.2 per 1000 catheter days, with a higher risk in children with SBS aged less than 1 year [48]. CRBSIs may be due to the contamination and improper care of the catheter, hub contamination or exit site infection with bacterial migration into the catheter, although they are mainly associated with the use of PN during the first month of life [49]. CRBIs are often associated with enteric bacteria in SBS patients, but this does not necessarily imply that they are due to bacterial translocation or from bacterial overgrowth in the small intestine.

The presence of CRBSI is demonstrated by positive culture from the catheter and/or positive peripheral culture. CRBSI and recurrent bacteremia can lead to a loss of vascular access, intestinal failure associated with liver disease and ultimately patient death [50]. Immediate evaluation and diagnosis, including the use of blood cultures and the early initiation of antibiotic therapy, is important. In relation to this, Hudgins et al. have shown that reducing the time to antibiotic therapy improves morbidity and mortality [20,51]. Fungal catheter-associated BSIs require central line removal and prolonged intravenous antifungal therapy [52]. 

The European Society of Paediatric Gastroenterology, Hepatology and Nutrition recommends that in every bowel rehabilitation center, there is a personalized strategy to prevent or recognize and treat CRBSI in the initial phase [53]. Some interventions, such as the routine replacement of central lines on a scheduled basis, antibiotic prophylaxis or the use of heparin, do not reduce the risk of infection [54,55]. Sufficient evidence has shown that the prophylactic use of an ethanol blockade reduces CRBSIs. If the patient is deemed to be at high risk or is otherwise destined for continuous PN after the first catheter-related infection, the technique of installing 70% ethanol in the catheter could be used, although catheter thrombosis may be a complication that needs to be addressed [50,56,57]. Oliviera et al. tested ethanol blocks in central venous catheters in their meta-analysis of four studies, demonstrating an 81% reduction in the risk of CRBSIs in children with IF [51]). In their review of the literature, Rahhal et al. reported observational studies demonstrating the efficacy and safety of the ethanol blockade compared with the standard heparin blockade [50]. Bradshaw et al. reported the role of taurolidine in reducing catheter-related sepsis [54]. However, the results of randomized controlled trials in adults appear to show the greater efficacy of a mixed taurolodine-citrate-heparin block compared to a simple taurolodine block for the prevention of CRBSI [58,59]. Furthermore, chlorhexidine-impregnated dressing appeared to be beneficial in preventing catheter colonization and CRBSI [60]. Strict disinfection during central line management by hospital staff and parents or healthcare professionals is important to minimize CRBSI occurrence [60].

### 7.2. Small Intestine Bacterial Overgrowth (SIBO)

Patients with SBS are prone to SIBO or increased numbers of bacteria in the small intestine [61]. Significant bowel resection causes the dilation of the remaining small bowel due to stenosis or ineffective propulsive waves. These factors, along with poor intestinal motility and inefficient nutrient absorption, promote SIBO.

The SIBO is defined as a growth >10^5^ CFU/mm^3^ of a bacterial species with the culture of luminal aspirate obtained by endoscopy [61,62]. Duodenal aspirate remains the gold standard for the diagnosis of SIBO [61,62]. The lactose or glucose breath test has been proposed for the diagnosis of SIBO, which is less invasive and expensive than the duodenal aspirate with a culture test that would be the gold standard [61,62]. According to the North American Consensus, the test is positive if lactulose or glucose for hydrogen are above the $20 ppm from the baseline within 90 min or for methane above the $10 ppm at any time during testing in association with a clinical manifestation suggestive of SIBO [63].

After a significant intestinal resection, the remaining intestine tends to increase its caliber due to ineffective stenosis or propulsive waves, whereby the dilation of the small intestine, poor intestinal motility, inefficient absorption of nutrients and loss of the ileocecal valve, together with dependence on PN and the suppression of gastric acidity, promote SIBO [62]. Clinically, SIBO manifests mainly with abdominal distension, bloating, meteorism with weight loss, faltering growth and the exacerbation of diarrhea and malodorous stools [64]. These symptoms of SIBO mainly result from nutrient malabsorption and can delay parenteral weaning [61,64] and trigger an inflammatory cascade, leading to the systemic distribution of bacterial antigen-antibody complexes that can cause skin rashes, arthritis, nephritis, endotoxemia and sepsis [65].

Related to SIBO, there will be a vitamin B12 deficiency given the role of bacteria in competing for dietary vitamin B12 [65]. Malabsorption aggravated by occult blood loss, vitamin B12 deficiency or both can cause anemia [65].

The main organisms responsible for SIBO include *Escherichia coli*, *Klebsiella* spp. and *Aeromonas* [66]. Other common species include *Streptococci* and *Bacteiroidetes* [66]. El Kasmi et al. also demonstrated in mouse models that the overgrowth of *Erysipelotrichaceae* and *Bacteroidetes* species caused cholestasis and liver injury by increased intestinal permeability and lipopolysaccharide absorption [67]. The suppression of the gut microbiota was achieved by the combination of four oral antibiotics, resulting in blocking the hepatic damage of hepatic macrophage activation [67]. This confirms that the increased intestinal permeability and absorption of the bacterial cell wall products promote bacterial translocation and bacteremia, and are able to activate the innate immune system and promote sepsis episodes leading to liver damage.

**Figure 2:**
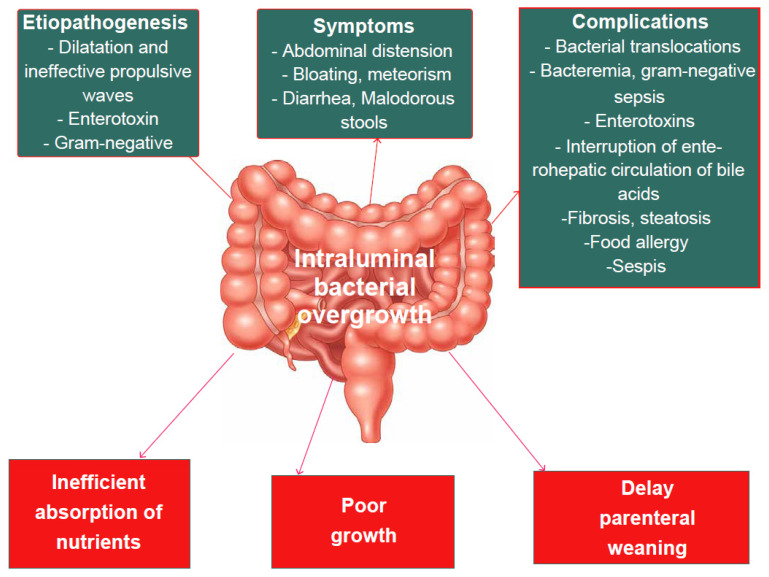
Consequences of small intestine bacterial overgrowth (SIBO).

Figure 2 summarizes the consequences of SIBO.

### 7.3. D-Lactic Acidosis

D-lactic acidosis is a rare but dramatic complication in SBS patients who have an intact colon in continuity with their small bowel. The production of D-lactate in patients with SBS is caused by an overgrowth of bacterial species, such as *Lactobacilli*, that are producers of both L-lactate and D-lactate, and by the lower absorption of carbohydrates that remain in the colon, which provide an energy substrate for anaerobic bacteria, leading to the production of lactate fermenting excess carbohydrates [68]. The lack of D-lactate dehydrogenase and D-lactate metabolism is slower than L-lactate metabolism. This can lead to SBS patients with D-lactate accumulation and metabolic acidosis having an increased anion gap [68].

Clinically, it is manifested by nuanced neurological symptoms: altered mental status weakness, gait disturbances, slurred speech and unexplained acidosis [69]. Laboratory lactic acid assays that solely measure L-lactic acid report a normal value, and D-lactic acid must be specifically measured. Thus, the diagnosis may be missed. The primary therapeutic strategy is carbohydrate reduction [70]. Probiotics and prebiotics have been used in the treatment, although their effectiveness is not yet clear [71].

### 7.4. Intestinal Failure-Associated Liver Disease (IFALD)

IFALD is defined by Lacaille et al. as a hepatobiliary dysfunction caused by the medical and surgical management of the intestinal insufficiency underlying SBS [53]. The diagnosis of IFALD is usually made on a clinical basis, associated with the presence of laboratory indices of cholestasis in children requiring long-term PN for SBS. In the IFALD, there is a history of cholestasis with laboratory indices of direct bilirubin ≥ 2 mg/dL for 2 consecutive weeks [72,73]. Severe IFALD and end-stage liver disease may be predicted by persistently elevated bilirubinemia values (>6 mg/dL) and the prolongation of prothrombin time [74,75]. Radiological examinations permit us to evaluate the associated splenomegaly in advanced liver disease and study the liver parenchyma [48]. In selected cases, the staging of the disease and the decision of the optimal therapeutic action takes place through a liver biopsy [48].

The pathogenesis of IFALD is multifactorial. Risk factors for the development of IFALD include prematurity, interruption of enterohepatic circulation of bile acids, intestinal stasis with bacterial proliferation and translocation, reduced portal venous return, leading to ischemia or decreased blood flow resulting in inflammation and intestinal necrosis, recurrent sepsis related to the central venous catheter, excessive glucose intake, which causes hyperinsulinism and subsequent steatosis, and long-term parenteral nutrition [14,76,77,78]. IFALD occurs in 40–60% of patients who remain on long-term PN [75,79]. It can progress and become intractable, compromising bowel rehabilitation and leading to liver and bowel transplantation [73]. IFALD can progress rapidly if early management is not provided to prevent progression to end-stage liver disease. Sepsis appears to be an independent predictor of the IFALD, and each septic episode is associated with a 3.2-fold increase in the risk of developing jaundice [46,80]. Gram-negative CRBSIs may increase hepatic dysfunction. Endotoxins released during sepsis have been shown to directly or indirectly affect bile excretion by acting on bile transport proteins, leading to cholestasis [81,82]. Prematurity is an established risk factor for IFALD [83]. Premature infants who develop necrotizing enterocolitis (NEC) are more likely to develop IFALD because they have multiple risk factors: short bowel, sepsis, intestinal obstruction, interrupted enterohepatic circulation of bile acids, high glucose and lipid intake and the need for continuous rather than cyclic PN infusion [53].

**Figure 3:**
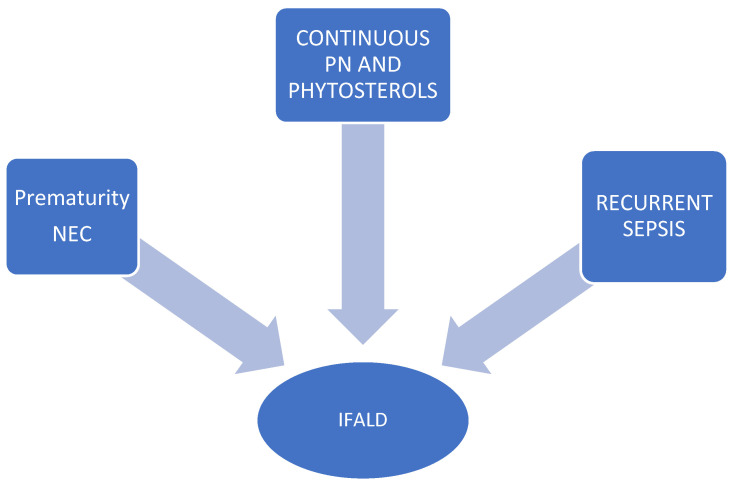
Main reasons for occurrence of intestinal failure-associated liver disease (IFALD) in children with short-bowel syndrome.

Children with SBS, due to their prolonged dependence on PN, are susceptible to the development of IFALD for the following reasons: (1) inappropriate use of lipid emulsions [46]; (2) presence of phytosterols in the lipid emulsion [84]; (3) administration of excessive amounts of glucose [85]; and (4) continuous instead of cyclic infusion duration [86]. Figure 3 summarizes main reasons for occurrence of IFALD in children with SBS.

### 7.5. Other Complications 

Patients with SBS are at risk of other chronic complications such as metabolic bone disease, renal and neurological dysfunction and a poor quality of life.

Metabolic bone diseases in children with SBS who undergo long-term PN range from osteopenia to severe bone disease with pathologic fractures [87,88]. The prevalence of these complications can be as high as 80% and are related to long-term PN, residual small bowel and inadequate vitamin and calcium supplementation [88,89]. Derepas et al. demonstrated that metabolic bone disease in children on long-term PN is mainly characterized by low bone synthesis with serum osteocalcin concentrations (i.e., a marker of bone formation) lower than in controls and higher in c-telopeptide (i.e., a marker of bone resorption) [89].

Renal disease in patients with SBS is multifactorial and includes chronic dehydration and electrolyte imbalances due to intestinal leakage from malabsorption, recurrent sepsis, nephrocalcinosis and the nephrotoxic effect of medications [90]. Pironi et al. reported that in patients with SBS, renal function is impaired in up to 54% of cases after intestinal transplantation [91]. Long-term PN and a short residual small bowel are associated with decreased renal function during and after weaning from PN [52]. It is therefore important to monitor the renal function of patients undergoing long-term PN. Billing et al. demonstrated that urinary α-1-microglobulin is associated with the duration of PN and is increased in SBS patients with chronic kidney disease, while serum creatinine and urea are not reliable markers because they remain normal in most patients until renal dysfunction is advanced [92]. One sign of possible renal disease could be abnormal echogenicity/nephrocalcinosis on ultrasound, which Ylinen et al. found in about half of pediatric patients with SBS at a mean age of 4 years [93]. 

Risk factors for neurological deterioration in SBS infants are preterm birth, long-term PN resulting in possible recurrent episodes of sepsis and prolonged hospitalizations with intensive medical and surgical treatments required in the first years of life [94]. Studies examining children with NEC have demonstrated delays in physical growth and cognitive and motor function [95,96]. Patients who undergo early surgery or have increased disease severity or additional bowel defects have an increased risk of microcephaly and cerebral palsy [94,96].

## 8. Nutritional Therapies

The cornerstones for the therapy of SBS associated with IF are meticulously administered fluids and personalized parenteral and enteral nutritional therapy [97]. Infants who have undergone massive bowel resection should receive parenteral nutrition early in the postoperative period, and EN should be initiated shortly thereafter. In order to set up proper parenteral and enteral nutritional therapy, it is necessary to consider: the cause of intestinal failure, the underlying nutritional status (considering prematurity, low birth weight or size appropriate for gestational age), gastrointestinal anatomy (length of the residue small and large intestine), vascular access, concomitant medical and surgical diseases, family medical history and social history [4].

### 8.1. Enteral Nutrition (EN)

EN promotes segment-specific compensatory changes that maintain the bowel absorptive function (i.e., the increase in small intestinal mucosal thickness, villus length, crypt depth) [98], ultimately decreasing the requirements of PN. Moreover, EN does not favor bacterial overgrowth [99]. The lack of enteral feeding impairs the enterohepatic circulation and bile acid secretion/absorption, leading to mucosal atrophy and increasing the risk of bacterial translocation. It has been demonstrated that the hormones stimulated by EN (e.g., motilin, glucose-dependent insulin tropic polypeptide, cholecystokinin, gastrin and glucagon) are decreased in patients on PN [25,99]. 

The composition and timing of enteral feeding can influence the achievement of enteral autonomy. Early onset of enteral feeding after the intestine resection has been reported to improve the rate of enteral autonomy [100]. Feeds should be increased gradually as tolerated. Tolerance is evaluated by measuring stool number and volume and by the observation of vomiting, irritability and abdominal as well as intestinal distension [7]. The management of SBS patients aims at promoting the bowel adaptation by using the gastrointestinal (GI) tract by oral feeding (OF) as much as possible, which is more physiological than enteral tube feeding (ETF). PN aims at promoting normal somatic growth during the time bridge for achieving intestinal autonomy. PN should not be stopped until adequate growth can be achieved with only OF and/or ETF. The stimulation of the hormones released by the GI tract promotes adaptation, whereas alternating fasting and feeding periods along with cyclical PN avoids the permanent secretion of insulin and excessive fat synthesis and deposition (i.e., steatosis and fat body mass) [7]. This suggested the important role of the very early oral feeding stimulation in preventing intestinal failure complications, particularly IFALD [101]. Another important reason to encourage it is to avoid food aversion that in the long-term may jeopardize the weaning of PN [102]. Forced continuous ETF may worsen fluid, minerals and nutrients malabsorption, and may result in severe perianal skin lesions [7]. Breast feeding should be encouraged. For infants with SBS, human milk is often chosen for EN, but data to support this choice are limited. Human milk contains growth factors, amino acids, immunoglobulins and other immunologically important compounds that may promote intestinal adaptation, and contains a number of factors supporting the developing neonate’s intestinal microbiota and immune system [103,104]. It has been hypothesized that the use of human milk may result in fewer days of dependence on PN [83] and may reduce the risk of IFALD [105]. Polymeric diets are not usually used, while extensively hydrolyzed formula (EHF) is preferred. The latter has the advantages of containing short peptides, better absorbed than free amino acids, as well as medium-chain triglycerides (MCT) [101]. The use of a MCT-rich diet (protein hydrolysate) by OF seems a good option [7]. The optimal strategy for enteral feeding, OF vs. ETF and bolus vs. continuous, remains debatable [101]. In children with chronic diarrhea, EN delivered by continuous drip has been shown to improve intestinal absorption and weight gain [106]. Bolus enteral feeding results in cyclical changes in plasma levels of gastrointestinal hormones, such as insulin, pancreatic polypeptide, gastric inhibitory polypeptide, gastrin, motilin, enteroglucagon and neurotensin, which may be important for adaptation and growth [107]. A combined approach (i.e., continuous feeding at night and bolus feeding during the day) is feasible [108]. The introduction of oral boluses of human milk or formula as soon as they are tolerated postoperatively appears to help stimulate oral motor development and may help to prevent long-term feeding aversion [109].

### 8.2. Micronutrient Supplementation

Micronutrient (vitamin and mineral) supplementation is a critical aspect of nutritional therapy: common nutrient deficiencies that develop in children with SBS include deficiencies of vitamin D, zinc, iron and vitamin B12; deficiencies of these and other nutrients can be observed even with the use of full or “total” PN, especially during the weaning from parenteral to enteral nutrition [110,111]. Enterally administered, water-soluble preparations of fat-soluble vitamins are helpful. In patients who have undergone terminal ileal resection, parenteral vitamin B12 may be necessary [4]. These patients should periodically undergo laboratory tests to identify any micronutrient deficiencies, which may be implemented as they can also be asymptomatic.

### 8.3. Parental Nutrition (PN)

PN has significantly improved the life expectancy of children with IF, defined as the reduction of the functional gut mass below the necessary amount to provide adequate nutrient and fluid requirements to warrant normal body growth in children [76]. In addition, the scientific advance in neonatal intensive care skills has led to a steep drop in the mortality of infants with SBS and IF [112]. Thus, the number of PN-dependent children has increased, and so has the morbidity [102]. The restoration of intestinal continuity should be done whenever possible as soon as possible. With improved colonic absorption, PN can then be discontinued or at least decreased. In addition, anastomosis enables the colonic fermentation of unabsorbed carbohydrates from the small intestine to occur, being an important source of energy assimilation [113].

Long-term PN administration is best achieved at home. Home PN, first used in the early 1980s, allows for the full nutritional support of children with IF at home [7,114,115]. The survival of children receiving prolonged PN depends mainly on the underlying diagnosis [116,117]. Home PN must be tailored to the single patient, maintaining the goal of counteracting the deleterious aspects of the disease [118]. The organization and follow-up of home PN is supposed to be shared between pediatric gastroenterology-nutrition teams and home care-giver companies according to the local facilities [7]. 

In addition to the role of CRSBIs in causing IFALD, in children with SBS receiving PN, it is extremely important to be careful with the use of intravenous lipid emulsions (ILE) because in these patients, studies suggested a link between ILE and IFALD development [119,120,121,122,123]. The use of soybean oil-based ILE in PN may represent a major culprit in the development of IFALD, so several factors should be taken into consideration when choosing an ILE for parenteral use: the ratio of ω-6/ω-3, the polyunsaturated fatty acids (PUFAs) content, the content in essential fatty acids (FAs), the amount of medium-chain triglycerides (MCTs), α-tocopherol and phytosterols. The probable detrimental effect (pro-inflammatory) of ω-6 FAs on liver function is provided by studies that showed fat emulsions based on pure fish oil (containing ω-3 FAs) being successful as rescue therapy in pediatric patients with SBS affected by severe liver disease [116,123,124,125]. Intravenous fat preparations enriched with ω-3 FAs has had a beneficial effect on the severity of IFALD and has reduced mortality rates among infants with SBS [126,127]. The evidence gathered on the beneficial effects of fish oil in these patients has led to its use in clinical practice. In North America, a pure fish oil-based lipid emulsion (Omegaven^®^) has been promoted as a unique emulsion to be available on the US market [7]. In Europe, it is possible to use a composite lipid emulsion containing a mixture of soybean oil (30%), coconut oil (30%), olive oil (25%), fish oil (15%) and 200 mg/L of α-tocopherol (SMOF-lipid^®^), which has an appropriate balance between ω-6/ω-3 PUFAs. Omegaven^®^ as a unique source of ILE may not be able to provide enough calories to sustain growth, while the combination of several types of oil by mixing soybean oil (rich in ω-6 FAs), olive oil (rich in monounsaturated Fas), coconut oil (rich in MCTs) and fish oil (rich in ω-3 Fas) appears to promote better growth while limiting hepatic toxicity [127]. Moreover, the lower essential fatty acid content of Omegaven^®^ compared to other fish oil-containing ILEs, such as SMOF-lipid^®^, gives further concern regarding its use as a sole lipid emulsion in children with low enteral tolerance [127].

Preparations with pure fish oil have been shown to be effective in reversing cholestasis [127,128,129]. Children in whom IFALD develops may have cholestasis. The involvement of such patients in open label studies of various agents strongly suggests that switching from an emulsion containing predominantly ω-6 FAs to one that contains predominantly ω-3 FAs, and that is also low in phytosterols, reduces cholestasis [127]. However, their long-term use as the sole source of lipids is debated [130]. The commonly recommended dose of intravenous fat emulsion for infants receiving parental nutrition is 1 g per kilogram per day in infants with severe gastrointestinal disease who are likely to require long-term PM, in an effort to reduce the incidence or severity of IFALD [131,132,133,134]. Essential fatty acid deficiency may occur if fat emulsion is administered at a level below 1 g per kg per day [131]. Regular clinical and biochemical monitoring (total FAs profile, including the ratio of triene to tetraene) is essential for children receiving restricted amounts of intravenous fat emulsion [4].

Summarizing all the available studies, PN is a life-saving therapy in patients with SBS and must be introduced early, at the same time, as bowel loss. According to the most recent studies, in an attempt to minimize liver damage, low-fat parenteral nutrition (less than 1 g/kg) supplemented with essential fatty acids (omega-3) should be adopted, and PN should be tailored to the needs of the individual patient. 

## 9. Pharmacotherapeutic Options

The medical management of SBS focuses on replacing electrolyte and nutrient losses, replacing lost fluid, limiting diarrhea and maintaining adequate body weight and growth percentiles. The ultimate goal of treatment is to allow the residual intestine to adapt to resume full EN [135,136].

### 9.1. Antimotility Agents and Bile Acid Binding Resins

Diarrhea, a debilitating complication of SBS, is mainly managed with antimotility agents such as loperamide, a µ-opioid receptor agonist, which is effective in increasing transit time by slowing intestinal motility. Loperamide can bind to bile acid-binding resins, which consequently reduce their activity [137].

Cholestyramine could also be used. It belongs to the category of bile acid binding resins and may be useful for binding bile salts in patients with residual colon and choleretic diarrhea [36]. Cholestyramine is a non-digestible anion exchange polymer that binds to bile acids in the colon, creating insoluble complexes which are then excreted [137].

Children with extensive ileal resections have a net loss of bile acids because more bile acids are excreted than can be replaced through hepatic synthesis. For these patients, bile acid binding resins may exacerbate steatorrhea and fat malabsorption and should be avoided [138].

### 9.2. Proton Pump Inhibitors (PPIs)

Gastric hypersecretion occurs in more than half of patients after bowel resection. Proton pump inhibitors (PPIs) are the first-line drugs in this case as they suppress gastric acid secretion [137]. Gastric hypersecretion is typically transient, but can last up to 12 months [138]. The optimal duration of postoperative antacid therapy in affected patients is unknown. Since there are data that suggest a link between acid blockade and infections of the gastrointestinal and respiratory tract, presumably including bacterial proliferation in the small intestine [139], patients need to be weaned from antacid therapy as soon as possible [4].

### 9.3. Antibiotics

In children with SBS, antibiotic therapy is the main therapeutic option not only for CRSBIs, but also for SIBO. The evidence for the use of antibiotics in SIBO have been limited to small clinical studies of a modest quality [140,141]. Consequently, there are no universally accepted treatment approaches. Because antibiotic use is associated with the development of resistant bacteria, adverse reactions and increased opportunistic infections such as *Clostridioides difficile*, it is necessary to objectively diagnose SIBO before considering antibiotic therapy [142]. Some clinical trials have evaluated certain classes of antibiotics. Tetracyclines have been used in the past, although these drugs have a low eradication rate because they have no direct activity against anaerobes and are ineffective against bacterioids [143]. Some authors have evaluated the therapeutic efficacy of nonabsorbable antibiotics, such as rifaximin and neomycin, to minimize the potential side effects of systemic antibiotics [144,145,146,147,148,149]. Rifaximin shows bactericidal action against both aerobes and anaerobes, such as *Bacterioids, Lactobacilli* and *Clostridia*. It exhibits less toxicity than other systemic antibiotics because its absorption is less than 0.1%. Cuoco et al. evaluated the efficacy of rifaximin followed by a 20-day course of probiotics in the treatment of SIBO, resulting in an eradication rate above 50% and improved gastrointestinal symptoms [145]. Attar et al. demonstrated a statistically significant improvement in the mean daily number of stool evacuations achieved with norfloxacin in comparison with amoxicillin-clavulanic acid [148]. Castiglione et al. demonstrated the efficacy of ciprofloxacin and metronidazole in the eradication of SIBO in patients with Crohn’s disease [149]. Metronidazole has been used with satisfactory results as an alternative to tetracycline. In a study of patients with blind loop syndrome, metronidazole showed greater therapeutic efficacy than rifaximin [147] (148). Neomycin, a nonabsorbable aminoglycoside, has been shown to have poor efficacy when used alone in SIBO [142].

Considering all the available data, there is no conclusive information on the most effective therapy that should be used in the treatment of SIBO. Treatment decisions should be individualized considering the risks of long-term antibiotic therapy and the possibility of SIBO recurrence.

### 9.4. Hormonal Pharmacologic Agents 

Another medical strategy in SBS children is the use of trophic factors, such as the growth hormone, insulin, and glucagon-like peptide 2 (GLP-2), to promote villous growth and thus decrease the need for PN and its side effects [102].

Teduglutide, a recombinant analogue of glucagon-like peptide-2 (GLP-2), is a hormonal pharmacological agent that facilitates intestinal adaptation and increases intestinal absorption [150]). GLP-2 is a naturally occurring hormone secreted in the distal ileum and colon by enteroendocrine cells, and it delays gastric emptying by inducing the epithelial proliferation of the small intestine, increases the surface area of the gut mucosa, improves gut-barrier function, upregulates nutrient absorption, increases intestinal blood flow and decreases bone resorption [151,152]. Recently, GLP-2 was suggested to have a direct effect on IFALD throughout the alteration of bile acid metabolism [153]. Patients with low levels of GLP-2 following the resection of the terminal ileum improved intestinal absorption and nutritional status after treatment with GLP-2 [154]. It is currently approved for children who are over 12 months of age with SBS and stable on PN after a period of post-surgery intestinal adaptation [155]. It improves intestinal absorption, increasing the depth of the crypt and the height of the villi in patients with SBS [156]. However, the effects are reversible and the therapeutic effects are lost when the drug is stopped. 

A few studies on rhGH alone (or in combination with glutamine) have been carried out in PN-dependent children with SBS. Despite some decrease in the PN requirements during treatment, these trials showed little benefit on mucosal absorption in the long term [157,158,159]. A recent 12-week, open-label, randomized study involving 42 children compared daily treatment with teduglutide, given at three dose levels (0.0125 mg per kilogram in 8 children, 0.025 mg per kilogram in 14 or 0.05 mg per kilogram in 15) with the standard of care (in 5 children) [150]. Treatment with teduglutide at a dose of 0.025 or 0.05 mg per kilogram was associated with a reduction in PN. Oral insulin has been shown to be beneficial in animal models and might be assessed very soon in children [160].

Finally, there is also interest in the use of other trophic factors, such as EGF and insulin-like growth factor-1, in children with IF and SBS [161].

### 9.5. Preparations Based on Bile Acids

Ursodeoxycholic acid (UDCA) is traditionally used in cholestatic liver disease to stimulate bile flow (choleresis) [25,102]. The American guidelines suggest that further research is needed to strengthen the recommendation of UDCA administration in IFALD [25]. Nevertheless, UDCA is commonly prescribed in children with IFALD at a dose of 15–20 mg/kg/day [102].

### 9.6. Probiotics 

Probiotics may play a role in improving intestinal adaptation in SBS. The function of probiotics is to improve the mucosal barrier through their adhesion to mucosal surfaces [162,163]. Probiotics act by: (1) inhibiting the attack of pathogenic bacteria; (2) promoting the secretion of factors that improve barrier integrity; and (3) immunomodulating on immune system cells [164,165].

Animal studies show that the restoration of the healthy microbiota occurs rapidly after antibiotic therapy when treated with probiotics [166]. Probiotics, by establishing normal commensals, may help the intestinal maturation process in SBS infants who are frequently exposed to antibiotics. Probiotics compete for nutrients and promote the production of antimicrobial molecules. Intestinal commensal organisms play a key role in intestinal maturation. The intestinal microbiota has been shown to play a role in the expression of genes involved in several intestinal functions, including absorption, mucosal barrier function, metabolism, angiogenesis and intestinal maturation [167]. By restoring the gut flora with commensal bacteria, probiotics also reduce antibiotic-associated diarrhea, which occurs due to the destruction of the gut microbiota and subsequent overgrowth of pathogenic bacteria, such as *C. difficile* [168]. 

Pathogenic bacteria can increase intestinal permeability through the disruption of tight junctions, leading to increased bacterial translocation and subsequent sepsis [169]. The most effective probiotics have been shown to be *Lactobacilli* and *Bifidobacterium*, which can directly suppress or kill pathogenic bacteria through the production of antibacterial molecules, including short-chain fatty acids (SCFAs), acetate and lactate, which lower luminal pH to inhibit pathogen growth, and bacteriocins, which attack the cell membranes of target bacteria [170,171]. SCFAs are derived from the fermentation of carbohydrates and fiber by probiotics, which reduces the ileal mucosal atrophy caused by PN therapy and increases the proliferation and decreases the apoptosis of mucosal epithelial cells [172,173]. *Lactobacillus rhamnosus* GG has also been shown to produce soluble proteins that promote intestinal epithelial cell growth and prevent cytokine-induced apoptosis. *Bifidobacterium* has been shown to produce an unidentified antimicrobial molecule that inhibits *E. coli, Klebsiella pneumoniae, Yersinia pseudotuberculosis, Staphylococcus aureus* and *Salmonella typhimurium* [170]. *Lactobacilli* and *Bifidobacteria* increase the production of total and pathogen-specific IgA in the intestinal mucosa without producing probiotic-specific IgA [174]. *Lactobacillus casei shirota* has been shown to increase natural killer cell activity [175]. 

Experimental studies in rat infants have demonstrated the downregulation of proinflammatory cytokine production in response to bacterial lipopolysaccharide (LPS) with *Lactobacillus rhamnosus* GG treatment by preventing LPS-induced necrosis in the intestinal mucosa [176]. Zhong showed through a meta-analysis that the addition of probiotics to basic therapy could decrease H2 concentration, decrease abdominal pain and decontaminate SIBO without preventing it [177]. Through the anti-inflammatory effect, probiotics may play this role in improving intestinal adaptation in SBS by promoting food tolerance and protecting the liver from further injury.

## 10. Conclusions

SBS is a complex condition of both surgical and medical interest. The complications, due to the inability of the intestine to absorb enough nutrients and fluid to support growth and neurodevelopment, make this syndrome a potentially life-threatening condition. The severity of the syndrome depends on the loss of a specific length or segment of bowel and on the morphologic and functional changes of the intestinal adaptation that improve the absorptive capacity [3,4]. The development of knowledge in clinical practice has led to a reduction in mortality and morbidity with a long-term follow-up. Diagnostic and therapeutic decisions are ideally made by a multidisciplinary team that includes neonatologists, pediatric surgeons, gastroenterologists, pediatricians, nutritionists and nurses. The rehabilitation approach includes a nutritional, medical and surgical evaluation [5,6]. The main objective is to identify early patients at risk of long-term PN dependence in order to avoid the most serious complications [23]. A significant improvement in prognosis can occur through the careful monitoring of nutritional status, avoiding dependence on PN and favoring an early introduction of EN, and through the prevention, diagnosis and aggressive treatment of CRSBIs and SIBO [42]. The outcome of pediatric patients with SBS has improved over the years, and intestinal transplantation should be considered as a therapeutic option when IF is irreversible and children develop severe complications related to long-term PN [76]. Multicenter initiatives, such as research consortium or data registries, are mandatory in order to personalize the management of these patients, improve their quality of life and reduce the cost of care.

## Data Availability

Not applicable.
